# Poly(ethylene Glycol) Methyl Ether Methacrylate-Based Injectable Hydrogels: Swelling, Rheological, and In Vitro Biocompatibility Properties with ATDC5 Chondrogenic Lineage

**DOI:** 10.3390/polym15244635

**Published:** 2023-12-07

**Authors:** Yousof Farrag, Djedjiga Ait Eldjoudi, Mariam Farrag, María González-Rodríguez, Clara Ruiz-Fernández, Alfonso Cordero, María Varela-García, Carlos Torrijos Pulpón, Rebeca Bouza, Francisca Lago, Jesus Pino, Carmen Alvarez-Lorenzo, Oreste Gualillo

**Affiliations:** 1 Servizo Galego de Saude (SERGAS) and Instituto de Investigación Sanitaria de Santiago (IDIS), Neuroendocrine Interactions in Rheumatology and Inflammatory Diseases (NEIRID Group), Santiago University Clinical Hospital, Building C, Travesía da Choupana S/N, 15706 Santiago de Compostela, Spain; djidji.aiteldjoudi@gmail.com (D.A.E.); mariam.r.farrag@gmail.com (M.F.); maria.gonzalez3112@gmail.com (M.G.-R.); clararf94@gmail.com (C.R.-F.); sitoalcorba@gmail.com (A.C.); merymodre@gmail.com (M.V.-G.); carlostorrijos00@gmail.com (C.T.P.); oreste.gualillo@sergas.es (O.G.); 2 Grupo de Polímeros, Departamento de Física y Ciencias de la Tierra, Escuela Universitaria Politécnica, Universidade da Coruña, Serantes, Avda. 19 de Febrero s/n, 15471 Ferrol, Spain; rebeca.bouza@udc.es; 3 Servizo Galego de Saude (SERGAS) and Instituto de Investigación Sanitaria de Santiago (IDIS), Molecular and Cellular Cardiology Lab, Research Laboratory 7, Santiago University Clinical Hospital, C, Travesía da Choupana S/N, 15706 Santiago de Compostela, Spain; francisca.lago.paz@sergas.es; 4 I+D Farma Group (GI-1645), Departamento de Farmacología, Farmacia y Tecnología Farmacéutica, Facultad de Farmacia, Instituto de Materiales (iMATUS), Health Research Institute of Santiago de Compostela (IDIS), Universidade de Santiago de Compostela, 15782 Santiago de Compostela, Spain; carmen.alvarez.lorenzo@usc.es

**Keywords:** biopolymers, injectable hydrogels, PEGMEM, DMAEM, biocompatibility

## Abstract

Here, we present the synthesis of a series of chemical homopolymeric and copolymeric injectable hydrogels based on polyethylene glycol methyl ether methacrylate (PEGMEM) alone or with 2-dimethylamino ethyl methacrylate (DMAEM). The objective of this study was to investigate how the modification of hydrogel components influences the swelling, rheological attributes, and in vitro biocompatibility of the hydrogels. The hydrogels’ networks were formed via free radical polymerization, as assured by ^1^H nuclear magnetic resonance spectroscopy (^1^H NMR). The swelling of the hydrogels directly correlated with the monomer and the catalyst amounts, in addition to the molecular weight of the monomer. Rheological analysis revealed that most of the synthesized hydrogels had viscoelastic and shear-thinning properties. The storage modulus and the viscosity increased by increasing the monomer and the crosslinker fraction but decreased by increasing the catalyst. MTT analysis showed no potential toxicity of the homopolymeric hydrogels, whereas the copolymeric hydrogels were toxic only at high DMEAM concentrations. The crosslinker polyethylene glycol dimethacrylate (PEGDMA) induced inflammation in ATDC5 cells, as detected by the significant increase in nitric oxide synthase type II activity. The results suggest a range of highly tunable homopolymeric and copolymeric hydrogels as candidates for cartilage regeneration.

## 1. Introduction

Hydrogels are three-dimensional polymeric networks of mainly hydrophilic macromolecules that can retain a large amount of water without dissolving [1]. The polymeric network can be formed through covalent bonding (chemical hydrogels) or through weaker and typically reversible linkage (physical hydrogels). Hydrogels are being studied and employed for a wide variety of biomedical applications, including insulin, gene, and drug delivery [2,3,4,5], tissue engineering [6], biosensor membranes [7], wound healing [8,9,10], and contact lenses [11,12]. One of the major advantages of hydrogels, which locates them at the center of scientific interest, is that they can be molecularly engineered to obtain desired physical properties. These properties mainly include swelling behavior, mechanical performance, and biocompatibility. This fine control of the hydrogels’ final properties can be achieved more easily along with less batch-to-batch variability with synthetic hydrogels rather than those of natural origin.

Injectable hydrogels, characterized by their tuned physicochemical properties for in situ injection, have gained growing interest as biomaterials that can be administrated with minimally invasive procedures [8]. These hydrogels are engineered to be administered in a liquid state, subsequently undergoing in situ formation of a 3D hydrogel network [13]. However, certain injectable hydrogels, especially those with shear-thinning properties, can be directly injected in gel state [14]. The pliable nature of injectable hydrogels mitigates the risk of mechanical damage to the surrounding tissue and enables them to fit and fill irregular cavities in the administration site. These features make for more convenient and comfortable treatment and reduce surgical complications, infection risk, recovery times, and overall treatment costs [15]. 

Injectable hydrogels can be used for wound healing, tissue engineering, and local and sustained delivery of drugs, cells, genes, growth factors, and other bioactive molecules [16,17,18]. They are extremely promising for cartilage tissue engineering and regeneration, particularly in musculoskeletal inflammatory diseases like osteoarthritis, rheumatoid arthritis, and intervertebral disk degeneration [19,20]. In these pathologies, tissues undergo progressive degeneration and loss of function over time, leading to persistent pain and physical disability. Injectable hydrogels can serve as a vehicle for the locally sustained release of anti-inflammatory drugs and/or growth factors, potentially alleviating symptoms and promoting tissue healing [21]. In addition, the lubrication properties of such materials can also help to restore, although temporarily, the cartilage rheological properties of the synovial fluid [19]. An example of enhancing the viscous properties of the synovia fluid using hydrogels is viscosupplementation, which is the intra-articular injection of hyaluronic acid in different formulations and molecular weights. It has been approved as a therapeutic alternative for the symptomatic management of osteoarthritis [22]. Researchers have explored different materials for their use as injectable hydrogels in the last few years, including both natural and synthetic hydrogels. Hyaluronic acid, chitosan, gelatin, alginate, silk fibroin, and carrageenan are examples of natural polymers used as injectable hydrogels, whereas PEG and poly(lactic-co-glycolic acid) (PLGA) are the most used polymers for synthetic hydrogels [8,23,24,25]. 

We have previously reported the preparation of composite biomaterials based on a copolymeric hydrogel synthesized of polyethylene glycol methyl ether methacrylate (PEGMEM) and 2-dimethylamino ethyl methacrylate (DMAEM) as monomers and N,N′-methylenebis(acrylamide) (BIS) as crosslinker [26]. These hydrogels were mineralized with calcium phosphate and had high mechanical properties as they were intended for bone regeneration. These hydrogels were further reinforced mechanically with different natural polymers forming different IPN hydrogels [27]. The current study investigates the synthesis of a series of homopolymeric and copolymeric injectable hydrogels based on PEGMEM and DMAEM for their use in cartilage regeneration. Both monomers have been reported to be used for several biomedical applications [27,28,29,30]; however, the safety of polymers based on DMAEM is still controversial [31,32,33]. The previously described synthesis procedure [26] was modified to obtain more flowable hydrogel to fulfill the injectability requirement. Through systematic alterations in the synthetic components, we aim to investigate the impact on crucial properties such as swelling behavior, rheological attributes, and in vitro biocompatibility. The ATDC5 cell lineage was used to assess the biocompatibility assays as a well-known, excellent chondrogenic in vitro model for evaluating any possible hydrogel-induced cytotoxicity or nitric oxide (NO) production [34,35].

## 2. Materials and Methods

### 2.1. Materials and Reagents

The monomers polyethylene glycol methyl ether methacrylate (PEGMEM), Mw: 300, 500, and 950 g·mol^−1^ and 2-dimethylamino ethyl methacrylate (DMAEM), the crosslinkers N,N′-methylenebis(acrylamide) (BIS) and polyethylene glycol dimethacrylate (PEGDMA), Mw: 550, and the catalyst N,N,N′,N′-Tetramethylethylenediamine (TMED) were purchased from Sigma Aldrich, Darmstadt, Germany. The initiator ammonium persulfate (APS) was purchased from Bio-Rad, Tokyo, Japan. All chemicals were used without further purification. The water used in the preparation and dialysis was purified on a Milli-Q ultrapure system (Millipore, Molsheim, France).

### 2.2. Synthesis of the Hydrogels

The hydrogels were prepared according to the method described before with modifications [26]. The measured quantity of the monomers or oligomers was added to 4 mL of deionized water at room temperature. Subsequently, the catalyst and the crosslinker were added in the appropriate amounts. The APS was dissolved in deionized water and then 1 mL of its solution was added dropwise under stirring to initiate the free radical polymerization. The mixture was allowed to polymerize overnight, and the temperature of the solution was recorded every 30 s during the first hour. After preparation, the hydrogels were dialyzed against milli-Q water at room temperature for 10 days to remove the unreacted monomers and oligomers [29]. The hydrogels were subsequently sterilized using UV for 1 h with a UV lamp with an average irradiance of 0.1 mW/cm^2^. All subsequent experiments were performed on the sterilized samples.

The amounts of monomers or oligomers, crosslinkers, catalysts, and the initiator were varied to obtain gels with adequate rheological properties and to explore the effect of varying each component on the properties and biocompatibility of the final hydrogel product. The compositions of all prepared hydrogels are detailed in Table 1.

### 2.3. ^1^H Nuclear Magnetic Resonance (^1^H NMR)

Hydrogels were freeze-dried (LyoQuest, Telstar, Terrassa, Spain), dispersed in deuterated water, and then analyzed in a Bruker Avance 500 spectrometer (frequency of ^1^H, 500.13 MHz) (Bremen, Germany). ^1^H spectra (sequence zg) were obtained, and chemical shifts were reported in parts per million (ppm) downfield relative to tetra-methylsilane (TMS, 0.0 ppm). The spectra were analyzed and plotted using TopSpin software (Bruker, Billerica, MD, USA).

### 2.4. Hydrogel Swelling Behavior

After polymerization, 5 mL of the prepared hydrogels was inserted in a dialysis tubing cellulose membrane (Sigma Aldrich, Taufkirchen, Germany) with a 14,000 Da molecular weight cut-off. The dialysis then was performed in 200 mL of deionized water at room temperature for 10 days with continuous stirring. The resulting swelled hydrogel was then collected in a graduated cylinder to determine the final volume.

The hydration of the hydrogels was calculated according to Equation (1).

(1) $$H=\frac{V_{f}-V_{i}}{V_{i}}$$

where *V_i_* and *V_f_* are the volumes of the gels before and after the dialysis, respectively.

### 2.5. Rheological Properties of the Hydrogels

For each rheological test, approximately 1 mL of the hydrogel was loaded into the rheometer (AR 1000-N, TA Instruments, New Castle, DE, USA) equipped with a cone plate geometry (4 cm diameter, 1.58° angle, 50 µm gap). First, the storage “elastic” (G′) and the loss “viscous” (G″) moduli were monitored at 25 °C during a frequency sweep at angular frequencies from 0.05 to 50 rad/s. The temperature was then elevated to 37 °C at a rate of 2 °C/min, and the frequency sweep was repeated within the same angular frequencies. All measurements were conducted at a strain of 0.1%, which was within the linear viscoelastic range of the material, as confirmed by a strain sweep and the absence of a third harmonic response.

### 2.6. Biocompatibility Assays

#### 2.6.1. Cell Culture

The murine chondrogenic cell line ATDC5 (RIKEN Cell Bank, Tsukuba, Japan) was cultured in DMEM/Ham’s F12 (Lonza Group Ltd., Basel, Switzerland) supplemented with 5% FBS (Merck KGaA, Darmstadt, Germany), 10 μg mL^−1^ human transferrin, 3 × 10^−8^ M sodium selenite, 4 mM L-glutamine, 50 units mL^−1^ penicillin, and 50 µg mL^−1^ streptomycin (Sigma-Aldrich, St. Louis, MI, USA) at 37 °C with 5% CO_2_ humidified atmosphere.

#### 2.6.2. MTT Viability Assay

The toxicity of the hydrogels was determined using the 3-(4,5-di- methylthiazol-2-yl)-2,5-diphenyltetrazolium bromide (MTT) (Sigma-Aldrich, USA) cell viability assay. MTT is a yellow compound that when reduced by living mitochondria, produces purple formazan crystals that, once solubilized, can be measured spectrophotometrically. The quantity of formazan is directly proportional to the number of viable cells and their metabolic activity. For this purpose, 8000 ATDC5 cells/well were seeded in a 96-well plate and left in culture media overnight. The culture media was then replaced by 100 µL of a mixture of 25% dialyzed hydrogel and 75% serum-free medium, and the cells were incubated for another 24 h. To assess cell viability, 10 μL of 0.5 mg mL^−1^ of MTT solution was added into each well, 4 h prior to completing the incubation period. At the end of the incubation, the formazan crystals were dissolved with 100 µL of a solution of 10% SDS solution in 0.01 M HCl. After overnight incubation at 37 °C, the absorbance at 550 nm was measured in a microplate reader (MultiscanEX, Thermo Fisher Scientific, Waltham, MA, USA). Data are represented as percentage of control (untreated cells).

#### 2.6.3. Nitrite Assay

The activity of nitric oxide synthase type II, a master enzyme involved in the inflammatory response, can be monitored in vitro by evaluating the amount nitrite stable metabolites of NO. Nitrite accumulation was measured in the culture medium by Griess reaction. Briefly, The ATDC5 cells were seeded in a 24-well plate at a density of 125,000 cells/well. After overnight starvation, the cells were exposed to a mixture of 25% dialyzed hydrogel and 75% serum-free culture media for 24 and 48 h. Following these periods, 50 μL of the supernatant culture medium was mixed with 50 μL of Griess reagent (equal volumes of 1% sulfanilamide in 5% phosphoric acid and 0.1% naphtylethylenediamine HCl). An absorbance at 550 nm was measured in a microplate reader (MultiscanEX, Thermo Fisher Scientific, USA). Fresh culture medium was used as blank. The amount of nitrite production was calculated from a sodium nitrite standard curve freshly prepared in culture medium. Lipopolysaccharide (LPS) 100 ng mL^−1^ (*E. coli* serotype O26:B6, Sigma-Aldrich, USA) was used as the positive control and culture medium from untreated cells as the negative control.

#### 2.6.4. Statistical Analysis

All experimental data were obtained from at least 3 independent experiments. Data are expressed as mean ± standard error of the mean (SEM). Statistical analysis was performed using the Wilcoxon signed-rank test, as implemented in Prism 8 (GraphPad Software Inc., San Diego, CA, USA). Values with *p* < 0.05 were considered statistically significant.

## 3. Results and Discussion

### 3.1. Synthesis of the Hydrogel Network

Hydrogels were synthesized by free radical polymerization in water, employing a redox initiation system involving APS as initiator and TEMED as a catalyst. Initially, APS dissociates into sulfate ions (SO_4_^2−^), while the catalytic action of TEMED then accelerates the formation of sulfate free radicals (SO_4_^•−^) from APS. These sulfate free radicals convert the methacrylate monomers to free radicals that in turn react with unreacted monomer molecules, starting the polymerization reaction. The ongoing reactions lead to the random elongation of polymeric chains of the hydrogels [36,37]. This redox reaction is exothermic, and the control of the temperature during the gel formation is important, particularly if the gel is intended for the encapsulation of thermolabile active molecules. The evolution of the temperature was followed during the polymerization of the hydrogels prepared with the maximum concentration of each component to determine the maximum temperature during polymerization (T_p_). The homopolymeric hydrogel synthesized with 3.33 mmol of PEGMEM of all used molecular weights and 5 μL of TMED (PTEM5) showed no increase in temperature during polymerization. An increase in monomer amount to 5 mmol (P5) increased T_p_ to 27.1 °C. It is worth noting that this T_p_ was reached 40 min after initiating the polymerization. Increasing the TMED amount to 50 μL (PTEM50) accelerated the reaction substantially, as a T_p_ of 26.2 °C was reached after 18.5 min. The copolymeric hydrogel P3D3TEM50 reached a T_p_ of 27.6 °C after only 9 min. The presence of a strong basic tertiary amino group in the DMAEM increases the reaction kinetics and can even eliminate the need to use any catalyst during the reaction [26]. The maximum registered T_p_ was 29.2 °C and corresponded to the copolymeric hydrogel P2.5D2.4PDM4 synthesized with 4% PEGDMA as crosslinker. The temperature during the polymerization of the hydrogel prepared with the crosslinker BIS at the same concentration (P2.5D2.4BIS4) reached only 26.4 °C. This slight increase in temperature of a maximum of 5 °C can be considered adequate for the entrapment of biomolecules or drugs.

**Figure 1:**
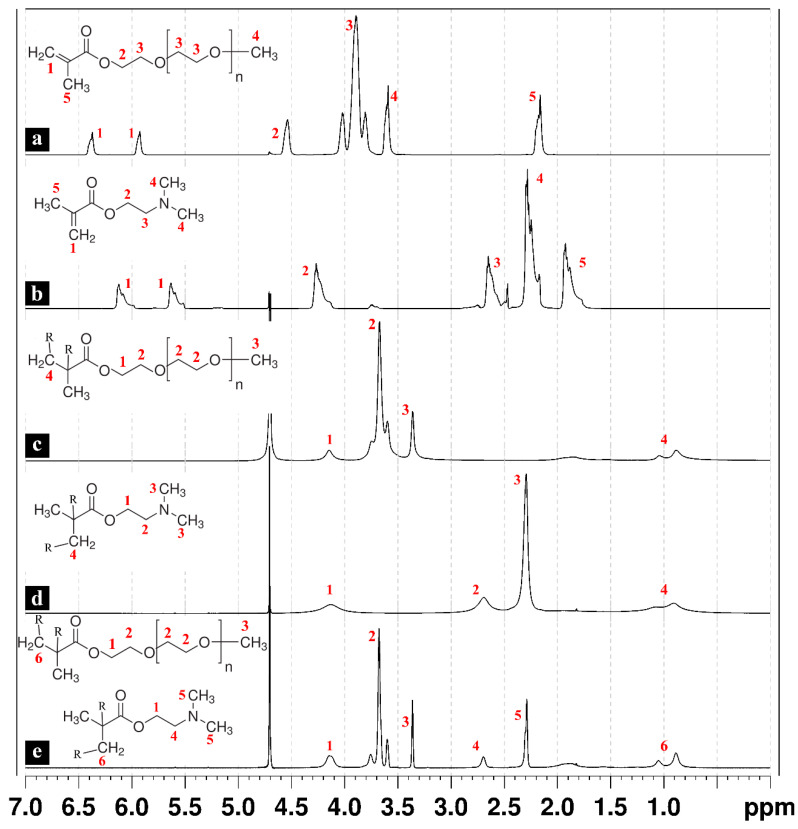
^1^H NMR spectra of the PEGMEM Mn = 300 (**a**) and DMAEM (**b**), poly(PEGMEM) (**c**), poly(DMAEM) (**d**), and the copolymeric hydrogel (**e**).

^1^H NMR spectra were recorded for the individual monomers in addition to the synthesized hydrogel polymers and copolymers. Figure 1a shows the typical spectrum of the PEGMEM, and the triplet between 3.8 and 4.0 and the peak at 4.6 are associated with the ethylene glycol units and the -OCO-CH_2_- attached to the methacrylate group, respectively. The peaks at 5.9 and 6.4 are associated with the two protons of the C=CH_2_ of the methacrylate group. In the case of using a PEGMEM of M_w_ = 300, the areas of the triplet at 3.8 to 4.0 and the peak at 4.6 are associated with a total of 18 hydrogens of the ethylene glycol units, meaning an average of four to five units in each oligomer molecule. The two peaks related to the -C=CH_2_ of the methacrylate group disappeared from the spectra of all hydrogels, which confirms the successful polymerization and the absence of methacrylate residues. The peaks corresponding to the -CH_2_- of the opened double bond appeared upfield at 0.88 to 1.04 (Figure 1c–e).

The ^1^H NMR of the DMAEM also shows the typical spectra, including the peaks at 5.6 and 6.1 related to the protons of the C=CH_2_ of the methacrylate group (Figure 1b) [38]. These two peaks also disappear from the spectra of the hydrogels synthesized by polymerizing only the DMAEM (Figure 1d).

The spectrum of the copolymeric hydrogel formed by both PEGMEM (Mw. 300) and DMAEM is presented in Figure 1e. The area of the peak at 2.29 is associated with six hydrogens of the two -CH_3_ groups attached to the nitrogen of the DMAEM. The area of the triplet at 3.59 and 3.75, which corresponds to 18 hydrogens, in addition to the area of the peak at 4.14 corresponding to four hydrogens, together represent 22 hydrogens of the PEG groups of the PEGMEM and the hydrogens of the -CH_2_-CH_2_-O of the DMAEM. These data indicated the proportion of the initial monomers in the final copolymer to be 1:1, which is the same equimolar ratio used in the synthesis [26].

### 3.2. Swelling Behavior

One of the important properties of hydrogels is their ability to absorb large amounts of water. During the swelling procedure, the synthesized hydrogels are also purified, removing the excess of unreacted reagents and the small-molecular-weight polymeric chains. The swelling behavior of the hydrogel has consequences on its properties and functionality, including the optical, surface, and mechanical properties, as well as the loading and release kinetics of nutrients, bioactive molecules, or cells, in addition to biodegradation [39]. The hydrophilic polymer network of the hydrogel acts as an osmotic membrane through which the osmotic pressure acts, leading to the swelling of the hydrogel until it reaches equilibrium. At equilibrium, the elastic energy of the polymeric chains balances the free energy of mixing the polymer chains with the solvent [40].

**Figure 2:**
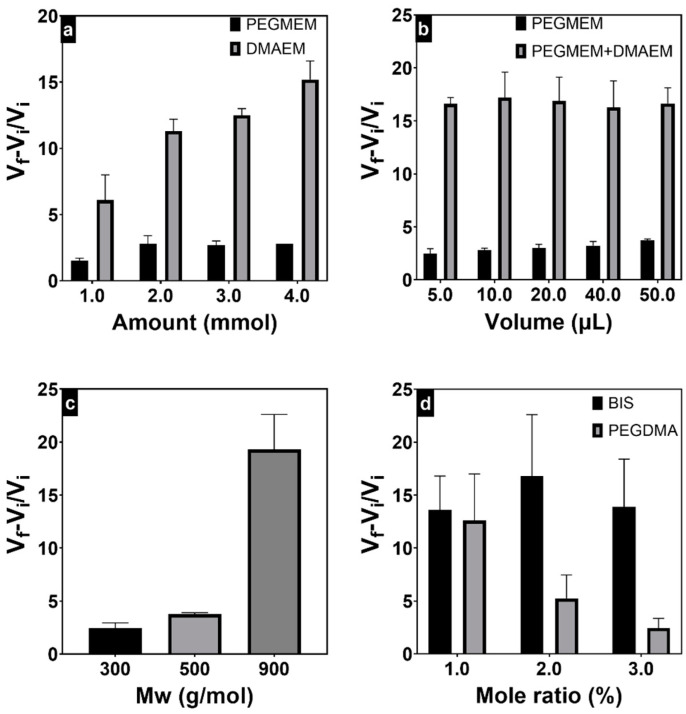
Hydration of hydrogels prepared with different amounts of monomers (**a**), amounts of catalyst TMED (**b**), PEGMEM molecular weights (**c**), and crosslinkers molar ratios (**d**).

The hydration of the hydrogels with different compositions was calculated by Equation (1) and presented in Figure 2. The increase in the amount of the PEGMEM 300 in the homopolymeric hydrogels from 1 mmol to 2 mmol almost doubled the swelling ratio from 1.5 to 2.8; however, higher oligomer amounts did not result in additional swelling. The use of PEGMEM with higher molecular weights increased the swelling ratio to 3.8 and 19.3 at molecular weights 500 and 900, respectively, (Figure 2c) due to the formation of a polymeric network with lower crosslinking density [41]. Regarding DMEAM hydrogels, a swelling ratio of 6.1 was observed at 1 mmol of DMAEM, and the swelling ratio increased by increasing the monomer amount, reaching 15.2 at 4 mmol. Given its cationic nature, the DMAEM homopolymeric hydrogels’ polymeric network presented increased hydration due to the charge repulsion among the polymer chains, thus augmenting the equilibrium swelling volume [42].

The swelling ratios of the PEGMEM homopolymeric hydrogels were higher when the polymerization was carried out with a greater amount of the catalyst TMED (Figure 2b). The higher catalyst amount resulted in the formation of shorter-length polymeric chains, creating a more flexible network that could absorb more water. Conversely, in copolymeric hydrogels, no noticeable change in swelling ratios was observed when increasing the TMED amount. This may be attributed to the possibility that shorter polymeric chains could decrease the repulsion forces between charged polymeric chains, potentially reducing the equilibrium swelling volume. The increase in PEGDMA crosslinker concentration decreased, as expected, the swelling ratios from 12.6 at 1% to 2.45 at a 3% molar ratio (Figure 2d). In contrast, the behavior of hydrogels crosslinked with BIS deviated from this pattern. This might be attributed to the cationic nature of BIS, which increased the hydration due to the charge repulsion among the polymer chains, counteracting the increased crosslinking density.

### 3.3. Rheological Characterization

**Figure 3:**
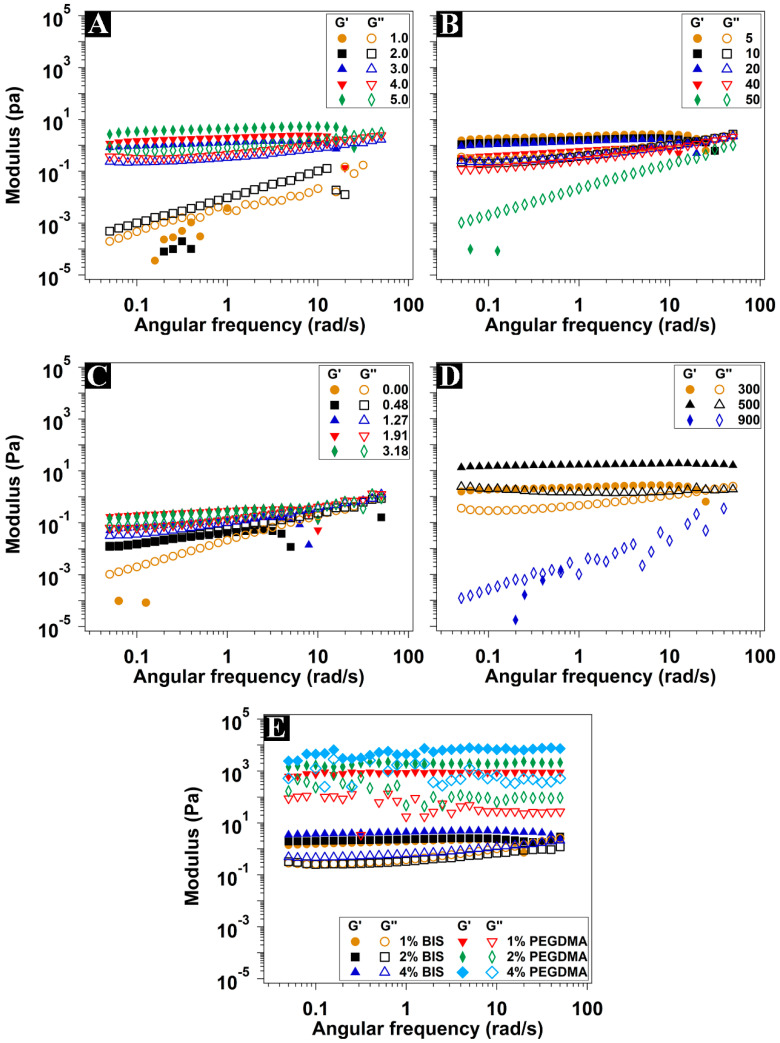
Elastic (G′) and viscous (G″) moduli of the hydrogels with different amounts of PEGMEM in mmol (**A**), TMED in µL (**B**), DMAEM in mmol (**C**), different molecular weights of PEGMEM (**D**), and crosslinkers in different concentrations in mole % to the monomers (**E**). Measurements were conducted within the linear viscoelastic region (LVR) and at a strain of 0.1%.

**Figure 4:**
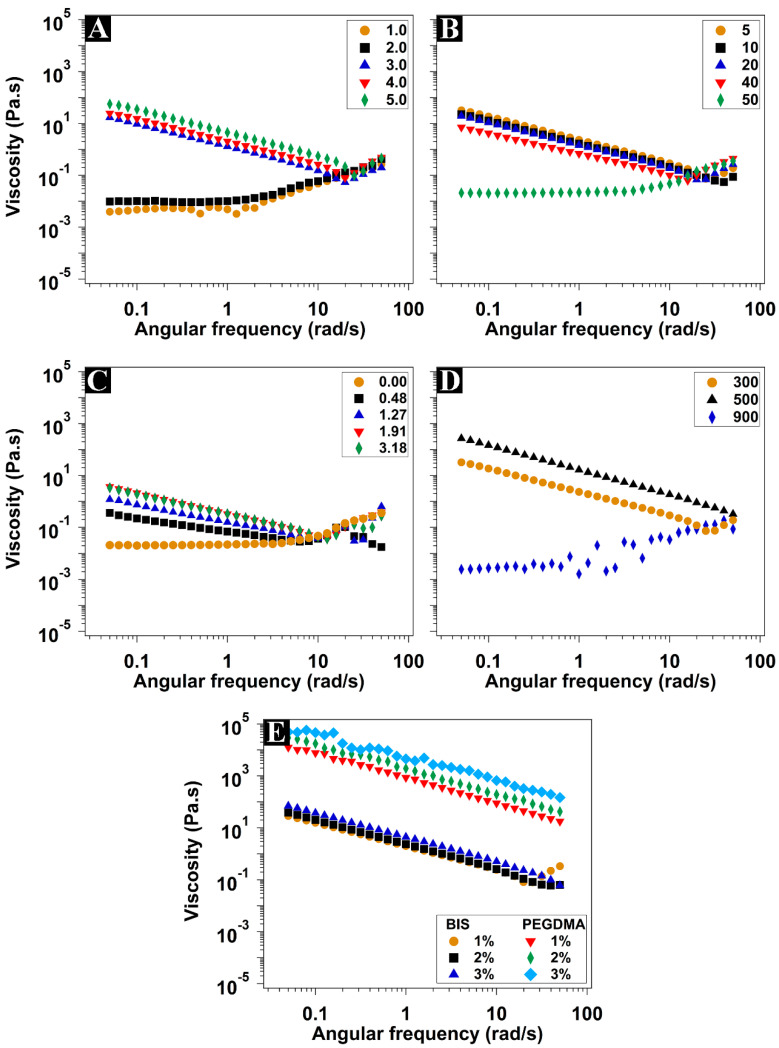
Complex viscosity (η*) of the hydrogels with different amounts of PEGMEM in mmol (**A**), TMED in µL (**B**), DMAEM in mmol (**C**), different molecular weights of PEGMEM (**D**), and crosslinkers in different concentrations in mole % to the monomers (**E**). Measurements were conducted within the linear viscoelastic region (LVR) and at a strain of 0.1%.

The rheological measurements were performed to approach an understanding of the effect of the different hydrogel components on the mechanical properties of the hydrogels. The elastic (storage G′) modulus, the viscous (loss G″) modulus, and the complex viscosity (η*) recorded at different shearing rates at physiological temperature (37 °C) are shown in Figure 3 and Figure 4. The elastic modulus represents the behavior of the solid component of the hydrogel (the energy stored during the application of the shear stress), while the viscous modulus represents the liquid behavior (the energy dissipated during the shear stress application) [43,44].

The hydrogels synthesized with up to 2 mmol of PEGMEM showed liquid-like properties, where G″ was dominating (Figure 3A). Moreover, these hydrogels showed shear-thickening properties, where the viscosity increased along with the shear rate (Figure 4A). However, at 3 mmol of PEGMEM and higher, the typical viscoelastic behavior of the hydrogels was clear where the G′ values were stable at all shear frequencies and were dominating over G″. In addition, there was a considerable increase in the viscosity, accompanied by shear-thinning characteristics. Both moduli, as well as the viscosity values, increased by increasing the monomer amount. The increase in monomer concentration led to a higher solid fraction in the synthesized hydrogels, thereby enhancing their elastic properties.

Hydrogels containing 3 mmol of PEGMEM were synthesized using TMED volumes ranging from 5 to 50 µL. The increase in the TMED amount led to a decrease in both G′ and G″, accompanied by a decrease in viscosity values. At the highest TMED amount used (50 µL), the obtained hydrogel exhibited liquid-like behavior, with dominating G″ and shear-thickening behavior (Figure 3B and Figure 4B). The use of more TMED eventually speeded up the polymerization, resulting in hydrogels characterized by shorter polymeric chains, lower viscosity, and weaker mechanical properties, resembling a liquid-like consistency.

The addition of 0.48 mmol of DMAEM to this hydrogel formula resulted in regaining the shear-thinning property (Figure 4C). On the other hand, the G′ was very slightly higher than the G″ at low frequencies until reaching the crossover point where G′ = G″ (Figure 3C). Passing the crossover point, the G″ was gradually dominating over the G’, which indicates liquid-like behavior. Moreover, G′ underwent a sharp decline beyond 3 rad/s. Increasing the DMAEM amount increased the values of G′, G″, and viscosity as well. It also shifted the crossover point along with the sudden decline in G′ to higher frequencies.

The use of PEGMEM of 500 g·mol^−1^ instead of 300 g·mol^−1^ enhanced both moduli and the viscosity of the hydrogel (Figure 3D). The small increase in PEG polymeric chain length was eventually responsible for the stiffer hydrogel, which maintained the shear-thinning property too. A further increase in the molecular weight to 900 g·mol^−1^ gave rise to a softer hydrogel with liquid-like properties, dominating G″ and shear-thickening properties, which is coherent with the swelling data previously discussed. The long PEG chains gave rise to a looser network, with a greater distance between adjacent crosslinking points, thus with lower stiffness. The decrease in G′ and G″ as a consequence of the greater swelling was also reported for multiblock segmented copolymeric hydrogel based on the PEG of different molecular weights and dimerized fatty acid [45].

The effect of the addition of different amounts of BIS or PEGDMA as crosslinkers on the rheological properties of copolymeric hydrogels prepared with equimolar amounts of PEGMEM and DMAEM is presented in Figure 3E and Figure 4E. All hydrogels had a dominating G′ with clear viscoelastic behavior and shear-thinning properties. Contrary to copolymeric hydrogel without crosslinkers, both G′ and G″ were independent on the applied shear frequency with no apparent crossover points or further decline in G′. The values of G′ and G″, as well as the complex viscosity of the hydrogels prepared with PEGDMA, were several orders of magnitude higher than those prepared with BIS. Increasing the concentration of both crosslinkers also increased G′, G″, and the viscosity values, with a more pronounced effect with PEGDMA. The higher stiffness of the hydrogels crosslinked with PEGDMA compared with those with BIS seems to also be related to their lower hydration and therefore higher solid fraction. The higher crosslinking degree as a result of using higher crosslinking concentration is expected to be accompanied by higher mechanical strength [46]. All tested hydrogels, except those crosslinked with PEGDMA, had moduli and viscosity values adequate for injectability through fine needles (more than 29 gauge) [47].

### 3.4. In Vitro Biocompatibility Assays

**Figure 5:**
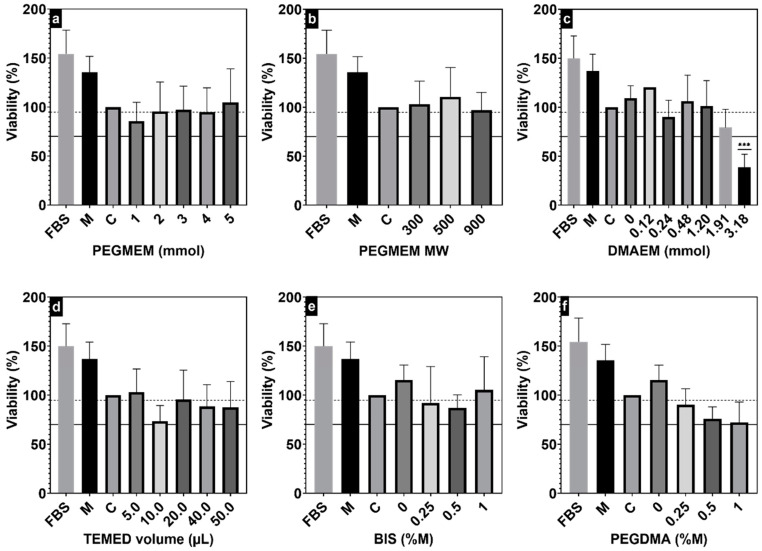
MTT assays showing the effect of PEGMEM amount (**a**) and MW (**b**), DMAEM amount (**c**), TMED volume (**d**), and the molar ratios of the crosslinkers BIS (**e**) and PEGDMA (**f**) on the viability of the ATDC5 cells. Results are expressed as mean ± SEM of at least three independent experiments. *** *p* < 0.001 against the control (C). The control (C) is 75% FBS-free culture medium and 25% water. The symbol (M) is for a second control of 100% FBS-free culture medium. Culture media with 5% FBS was used as positive control (FBS). A solid black line highlights the cytotoxicity threshold, while a dotted line highlights the 70% viability threshold calculated from M.

MTT assay was performed to evaluate any potential cytotoxic effects induced by the synthesized hydrogels (Figure 5). During the MTT assays, the different hydrogels were added to the ATDC5 culture medium at a concentration of 25% *v*/*v*. Given that most of the hydrogel composition is actually water, a diluted culture medium that contains 25% *v*/*v* water was used as the main control (C). When using PEGMEM as the only monomer, all synthesized hydrogels demonstrated cell viability exceeding the established 70% threshold (Figure 5a,b,d) [27,48]. When using the PEGDMA as crosslinker, there was a noticeable decrease in the viability of the ATDC5 cells; however, these decreases were not statistically significant (Figure 5f). Hydrogels synthesized using both PEGMEM and DMAEM did not exhibit any cytotoxic effect up to 1.2 mmol of DMAEM (Figure 5c). At 1.91 mmol of DMAEM, there was a noticeable, although statistically nonsignificant, decrease in the viability of the cells. Contrarily, hydrogels synthesized with 3.18 mmol of DMAEM were highly cytotoxic for ATDC5 cells, leading to viability values below 40%. Different hydrogel systems based on both monomers, even at higher concentrations, have been reported to be biocompatible with other cell lines, such as mouse monocyte macrophages (J774A.1) and murine preosteoblast cells (MC-3T3) [27,31]. DMAEM is a charged cationic monomer, which can be the reason for its potential cytotoxicity at higher concentrations. For further confirmation, a series of hydrogels were synthesized in a range of 1 to 4 mmol of DMAEM, 20 µL TMED, and 0.75 mg of APS, without any PEGMEM. All these hydrogels were extremely cytotoxic for the ATDC5 cells, showing microscopical features of cell death after only a few hours of incubation with the hydrogels.

**Figure 6:**
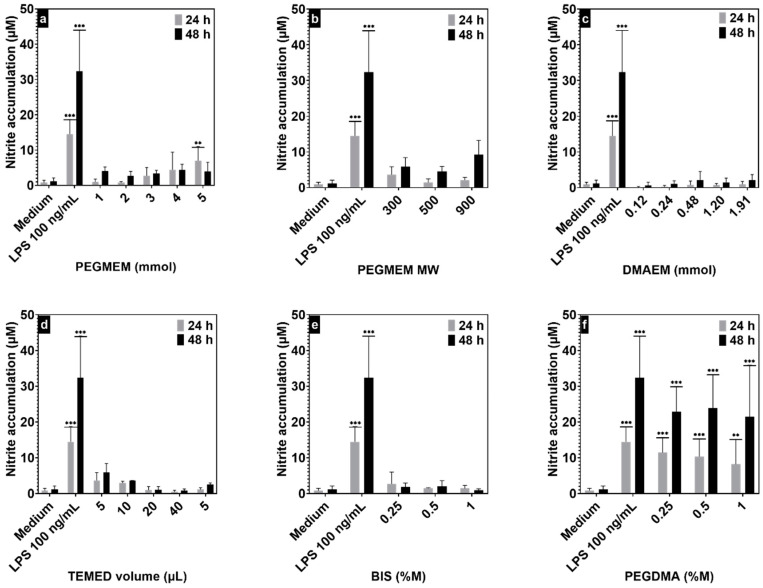
Nitrite accumulation at 24 h and 48 h of incubating the ATDC5 cells with hydrogels with different formulations showing the effect of PEGMEM amount (**a**) and MW (**b**), DMAEM amount (**c**), TMED volume (**d**), and the molar ratios of the crosslinkers BIS (**e**) and PEGDMA (**f**) on the viability of the ATDC5 cells. Results are expressed as mean ± SEM of at least three independent experiments. ** *p* < 0.01; *** *p* < 0.001. LPS 100 ng mL^−1^ was used as positive control and culture medium from untreated cells as negative control.

A nitrite assay was carried out to evaluate whether any of the components of the hydrogels have a potential proinflammatory effect (Figure 6). Inflammation is evaluated by the determination of the nitrite accumulation as a stable metabolite of NO, the endogenous gaseous mediator produced predominantly by nitric oxide synthase type II in chondrocytes in response to proinflammatory stimuli or injury. NO itself promotes cartilage damage by inducing chondrocyte apoptosis, matrix metalloproteinase synthesis, and proinflammatory cytokines expression [34]. Hydrogels synthesized with PEGMEM as the only monomer at different concentrations, different TMED amounts, or different molecular weights did not induce a significant increase in nitrite production compared with the control at both 24 and 48 h (Figure 6a,b,d).

Similarly, the copolymeric hydrogels did not significantly induce NO production, suggesting biocompatibility in chondrocytes. While there was no noticeable inflammatory response when using BIS as crosslinker, hydrogels crosslinked with PEGDMA had strong proinflammatory activity (Figure 5e,f).

## 4. Conclusions

We synthesized a series of homopolymeric and copolymeric injectable hydrogels with adjustable swelling and rheological properties. The incorporation of the neutral monomer PEGMEM and the cationic DMAEM allowed for the fine-tuning of hydrogel characteristics, affecting swelling, viscosity, shear-thinning behavior, and elasticity. The increased concentration of the catalyst TMED increased the swelling of the homopolymeric hydrogels, which in turn negatively affected their mechanical strength. The use of PEGDMA as a crosslinker at the concentration tested so far limited the injectability of the copolymeric hydrogels as it increased viscosity. The obtained results from the in vitro biocompatibility assays bring into question the biocompatibility of the crosslinker PEGDMA for cartilage regeneration applications as it induced the inflammation of ATDC5 cells at low concentrations. High concentrations of DMAEM were cytotoxic to these cells too. Conversely, PEGMEM-based homopolymeric hydrogels and copolymeric hydrogels with low DMEAM concentrations are potentially suitable candidates as injectable hydrogels with adequate mechanical properties for cartilage regeneration applications. They can also be applied to other clinical cases, such as IVDD and mandibular reconstitution surgeries. In addition, these hydrogels can be used as delivery systems for therapeutic agents, such as bioactive molecules, cells, and monoclonal antibodies. However, it is essential to note that the biocompatibility assays in this study are limited to osteoarthritis in vitro models. Additional in vivo biocompatibility and biodegradability testing are essential to validate and strengthen the proposed application of these hydrogels.

## Figures and Tables

**Table 1:** Composition of the different synthetic hydrogels.

Hydrogel ID	Water	PEGMEM MW and Amount			DMAEM Amount		Crosslinker			TMED Amount
	(mL)	MW	(mL)	(mmol)	(mL)	(mmol)	Type	Molar Ratio (M %)	(mg)	(µL)
**P1**	4	300	0.3	1.00	--	--	--	0.00	0.00	20.0
**P2**			0.6	2.00						20.0
**P3**			0.9	3.00						20.0
**P4**			1.2	4.00						20.0
**P5**			1.5	5.00						20.0
**PTEM5**			1	3.33						5.0
**PTEM10**			1	3.33						10.0
**PTEM20**			1	3.33						20.0
**PTEM40**			1	3.33						40.0
**PTEM50**			1	3.33						50.0
**P3MW500**	3.33	500	1.67	3.34						5.0
**P3MW900**	1	900	3	3.33						5.0
**P3BIS025**	4	300	1	3.33			BIS	0.25	1.3	5.0
**P3BIS05**			1	3.33				0.5	2.6	5.0
**P3BIS1**			1	3.33				1	5.1	5.0
**P3PDM025**			1	3.33			PEGDMA	0.25	4.6	5.0
**P3PDM05**			1	3.33				0.5	9.2	5.0
**P3PDM1**			1	3.33				1	18.3	5.0
**P3D024**			1	3.33	0.038	0.24	--	0.00	0.00	50.0
**P3D048**			1	3.33	0.075	0.48				50.0
**P3D127**			1	3.33	0.2	1.27				50.0
**P3D191**			1	3.33	0.3	1.91				50.0
**P13D3TEM5**			1	3.33	0.5	3.18				5.0
**P3D3TEM10**			1	3.33	0.5	3.18				10.0
**P3D3TEM20**			1	3.33	0.5	3.18				20.0
**P3D3TEM40**			1	3.33	0.5	3.18				40.0
**P3D3TEM50**			1	3.33	0.5	3.18				50.0
**P2.5D2.4BIS1**			0.75	2.50	0.375	2.39	BIS	1	7.5	--
**P2.5D2.4BIS2**			0.75	2.50	0.375	2.39		2	15.1	
**P2.5D2.4BIS4**			0.75	2.50	0.375	2.39		4	30.1	
**P2.5D2.4PDM1**			0.75	2.50	0.375	2.39	PEGDMA	1	26.9	
**P2.5D2.4PDM2**			0.75	2.50	0.375	2.39		2	53.7	
**P2.5D2.4PDM4**			0.75	2.50	0.375	2.39		4	107.5	

## Data Availability

Data are contained within the article.
